# Structural Organization of the 19S Proteasome Lid: Insights from MS of Intact Complexes

**DOI:** 10.1371/journal.pbio.0040267

**Published:** 2006-08-01

**Authors:** Michal Sharon, Thomas Taverner, Xavier I Ambroggio, Raymond J Deshaies, Carol V Robinson

**Affiliations:** **1**Department of Chemistry, University of Cambridge, Cambridge, United Kingdom; **2**Division of Biology, California Institute of Technology, Pasadena, California, United States of America; Biology Department, Technion-Israel Institute of TechnologyIsrael

## Abstract

The 26S proteasome contains a 19S regulatory particle that selects and unfolds ubiquitinated substrates for degradation in the 20S catalytic particle. To date there are no high-resolution structures of the 19S assembly, nor of the lid or base subcomplexes that constitute the 19S. Mass spectra of the intact lid complex from Saccharomyces cerevisiae show that eight of the nine subunits are present stoichiometrically and that a stable tetrameric subcomplex forms in solution. Application of tandem mass spectrometry to the intact lid complex reveals the subunit architecture, while the coupling of a cross-linking approach identifies further interaction partners. Taking together our results with previous analyses we are able to construct a comprehensive interaction map. In summary, our findings allow us to identify a scaffold for the assembly of the particle and to propose a regulatory mechanism that prevents exposure of the active site until assembly is complete. More generally, the results highlight the potential of mass spectrometry to add crucial insight into the structural organization of an endogenous, wild-type complex.

## Abstract 2

Tandem mass spectrometry coupled with structural cross-linking enabled a depiction of how components of the 19S proteasome lid interact and the active site remains hidden until assembly is complete.

## Introduction

In eukaryotes, the ubiquitin–proteasome pathway is essential for eliminating damaged or misfolded proteins and for the degradation of short-lived regulatory proteins [ 1, 2]. The two major subcomplexes of the 26S proteasome are the 20S proteolytic core particle (∼700 kDa) and the 19S regulatory particle (∼900 kDa). The 19S is attached at either or both ends of the 20S [ 3]. Selection of proteasomal substrates by the 19S regulatory particle occurs via the recognition of polyubiquitin chains bound to proteins that are destined to be degraded [ 4]. After selection of the labeled substrate, the polyubiquitin chain is then removed by the deubiquitinating enzymes Rpn11 [ 5, 6] and Ubp6 [ 7]. The substrate is then unfolded and translocated into the 20S channel, where it is degraded.

Nineteen different subunits have been identified in the 19S proteasome from yeast as well as mammals [ 8– 10]. This regulatory particle can be dissociated further into two subcomplexes, the base that binds directly to the 20S and a peripheral lid [ 11]. The base consists of six AAA-ATPase subunits, Rpt1–Rpt6, and four non-ATPase subunits, Rpn1, Rpn2, Rpn10, and Rpn13. The ATPases in the base are required for unfolding of substrate proteins [ 12] and channel opening [ 13] before translocation of substrate into the 20S cavity. The lid is composed of nine non-ATPase subunits: Rpn3, Rpn5–Rpn9, Rpn11, Rpn12, and Sem1. The major activity of the lid is proposed to be deubiquitination [ 5– 7]. Rpn10 and additional ubiquitin receptor proteins that reversibly associate with the proteasome, such as Rad23 and Dsk2, deliver the ubiquitinated protein to the proteasome [ 14].

The proteasome lid subunits exhibit high homology to the COP9 signalosome complex (CSN) [ 11], an essential regulator of diverse cellular and developmental processes, and has been found in most eukaryotic organisms ranging from yeast to human (for reviews see [ 15– 17]). The CSN contains eight core subunits that assemble into a 450-kDa particle. The subunits show a remarkable one-to-one sequence correspondence with those of the 19S lid, suggesting a common ancestry [ 11]. Of the lid components, Rpn11 is the most highly conserved subunit, with 65% identity between yeast and human proteins [ 6]. Recently it was identified as a Zn ^2+^-dependent metalloprotease responsible for substrate deubiquitination during proteasomal degradation [ 5, 6, 18, 19].

Although in the last few years functional characterization of the 19S lid has progressed considerably, the assembly poses a considerable challenge to structural biologists. High-resolution structural analyses are difficult since the complex is assembled in vivo and different subunits are expected to be dynamic and heterogeneous [ 20]. Only a low-resolution 3-D image of the 26S has been observed by electron microscopy [ 3], and the structural features of the lid complex were difficult to discern. Pairwise interactions among the lid subunits have been studied using the yeast two-hybrid system [ 20– 26]. Insight into the organization of the lid has also been gleaned from analysis of lid subcomplexes in cells that express mutant forms of specific lid subunits [ 25, 26]. However, a comprehensive model of subunit interactions within the lid has not been proposed, and no distinctive topological features, such as rings or well-defined channels, have been identified.

In this study, we aim to obtain novel information regarding the structural organization of the 19S lid from *Saccharomyces cerevisiae* using both tandem mass spectrometry (MS/MS) of the intact nine-component complex together with chemical cross-linking and MS analysis. It is established that MS and MS/MS can be applied to the study of intact noncovalent complexes, but to date, the majority of examples were of various complexes reconstituted in vitro [ 27– 29]. While some studies have been carried out on complexes isolated directly from cells, including bacterial RNA polymerase [ 30], ribosomes [ 31, 32], and RNA-processing complexes from yeast [ 33], existing structural information aided the interpretation of the resulting MS and MS/MS spectra. Here we show that the endogenous wild-type complex can be studied directly, without the need to generate mutants, and the results incorporated with existing yeast two-hybrid data to yield a comprehensive model. This represents a significant advance in MS methodology given the absence of existing high-resolution structural data. Moreover, the fact that all directly or indirectly interacting subunits can be observed within a single spectrum highlights one of the fundamental advantages of our methodology.

Using this approach the mass spectrum revealed not only the intact complex but also a substoichiometric complex. In addition, we identified the presence of an independent subcomplex containing Rpn5, Rpn6, Rpn8, and Rpn9, wherein Rpn8 occupies a central position. We could also demonstrate that Rpn6, Rpn9, and Rpn12 are found on the periphery of the complex. Complementary information from cross-linking approaches enabled us to show that Rpn5 binds to Rpn3. In addition, we could determine that the recently identified lid subunit Sem1 [ 9] binds to both Rpn7 and Rpn3. Taken together, our data allow us to propose a comprehensive model for the subunit organization of the 19S lid that incorporates our findings from MS with those of prior analyses of pairwise interactions.

## Results

### Electrospray Ionization MS and MS/MS of the Intact Lid

The electrospray mass spectrum of the intact 19S lid isolated from S. cerevisiae is shown in Figure 1. Despite the fact that the sample analyzed was the endogenous complex and was not overexpressed, the spectrum is well resolved and has one major series of peaks centered at 8,000 m/z. This major charge state series corresponds to the nine protein components of the lid, demonstrating clearly that all subunits must be interacting, either directly or indirectly, and present at unit stoichiometry. The measured mass of the intact complex is 376,151 ± 369 Da ( Table 1). Interestingly, an overlapping less-intense charge state series discerned at 7,750 m/z, indicates the existence of a substoichiometric complex that results from the absence of a subunit from the complex. Calculation of the mass difference of these series indicates a mass that is 50,270 Da less than that of the intact lid. This corresponds to the absence of Rpn6 from the intact lid. The similarity in the number of positive charges between the two complexes suggests that both complexes are present in solution (see below). An additional minor series of peaks is centered at 5,500 m/z. The calculated mass for this lower m/z charge series is 185,556 ± 137 Da, indicating that a smaller subcomplex of the lid is also present in solution.

**Figure 1:**
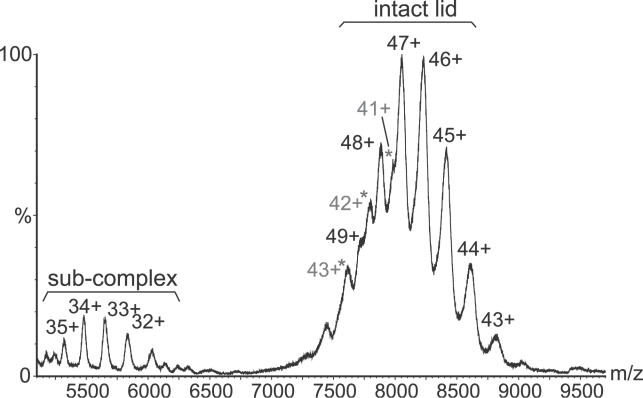
Electrospray Mass Spectrum of the 19S Lid Complex Isolated from S. cerevisiae The intact lid complex is observed between 7,250 and 9,000 m/z, with the most intense peak at ≈8,000 m/z. Well-resolved charge states are observed, and the measured mass confirms the presence of all nine subunits. An overlapping charge series is detected and corresponds to a substoichiometric complex in which the Rpn6 subunit is absent (labeled with asterisks). A subcomplex of the lid gives rise to a signal in the range of 5,000–6,250 m/z.

**Table 1:**
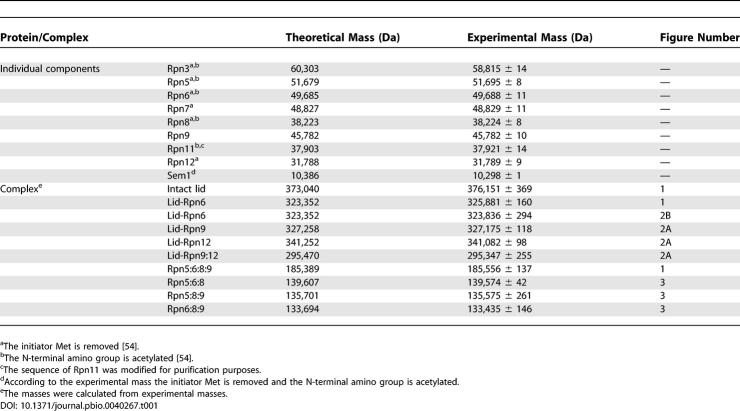
Theoretical and Measured Masses of Proteins and Complexes

To probe the composition and subunit organization of the lid we used an MS/MS approach [ 31, 34]. In such experiments, a specific well-defined m/z range that encompasses the parent ion is selected and accelerated through a collision cell at increased argon pressures. This process gives rise to multiple collisions in which the internal energy of the ions is accumulated. When this energy reaches a threshold value, dissociation occurs, yielding product ions. It is established that for noncovalent assemblies, individual highly charged subunits are dissociated from the parent ion, producing “stripped” complexes with lower charge states than the original ion [ 35– 38]. As a consequence, we can distinguish complexes formed in solution from those generated in the gas phase since the former will have charge states commensurate with their mass [ 39], while those formed in the gas phase will have anomalously low charge states. If we consider the propensity for dissociation of different protein subunits in the gas phase, it is known that under vacuum ionic interactions are enhanced, while hydrophobic interactions are weakened [ 40, 41]. However, we cannot rule out the effect of other factors that govern protein–protein interactions such as contact area, planarity, shape complementarity, etc. There is, however, a growing number of studies demonstrating that individual proteins that are expelled during tandem MS are those that are known from high-resolution structures to be more exposed and peripheral, while those at the core of the assembly are retained in the “stripped” complex [ 30, 32, 42, 43].

Applying this tandem MS strategy to the 376-kDa lid complex, using a collision cell acceleration voltage of 100 V, five series of ions were formed ( Figure 2A). At m/z values below 2,400, two highly charged series are observed. Their measured masses correspond to 45,782 ± 4 and 31,796 ± 10 Da, in very close agreement with the mass calculated for Rpn9 and Rpn12, respectively. At high m/z values between 10,000 and 18,500 three further charge series are observed. Their masses are consistent with the lid stripped of Rpn9 and/or Rpn12. These results indicate that both Rpn9 and Rpn12 have weaker interactions with other subunits and are peripheral, and hence are first to dissociate. Increasing the collision cell voltage, and hence the internal energy of the lid complex, induces the dissociation of an additional subunit ( Figure 2B). The new charge series observed at 2,000–2,400 m/z is assigned to Rpn6, and the corresponding stripped complex appears between 13,000 to 17,000 m/z. These results enable us to deduce therefore that Rpn6, Rpn9, and Rpn12 interact weakly and are presumably at the periphery of the lid subcomplex.

**Figure 2:**
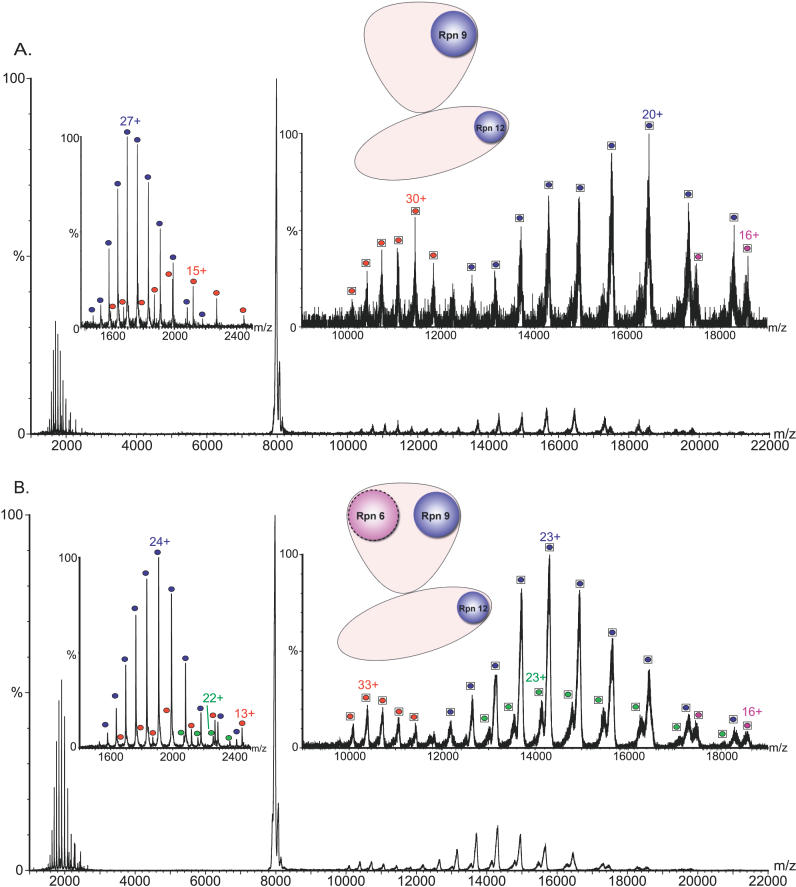
MS/MS of the 47+ Charge State of the Intact 19S Lid (A) Applying a voltage of 100 V to the collision cell generates product ions from the loss of only Rpn9 and/or Rpn12. Peaks between 12,500–17,000 m/z correspond to the loss of Rpn9 (blue squares), while the series at 10,000–12,000 m/z correspond to the loss of Rpn12 (red squares). The low intensity charge series at 17,000–18,000 m/z indicate the loss of both Rpn9 and Rpn12 (magenta squares). At low m/z 1,000–2,400 series of peaks are assigned to individual Rpn9 (blue circles) and Rpn12 (red circles). The data indicate the Rpn9 and Rpn12 have a relatively small contact surface with the other subunits, which implies that they are exposed. (B) Increasing the voltage to 120 V induces the dissociation of Rpn6 in addition to Rpn9 and Rpn12. The highly charged Rpn6 series is centered at ∼ 2,200 m/z (green circles), while the remaining stripped complex is observed between 13,000 to 18,000 m/z (green squares). In summary, Rpn9 and Rpn12 are easily dissociated from the lid complex (blue). An increase in the collision cell voltage also induces dissociation of Rpn6 (purple).

### Tandem Mass Spectrum of the Lid Subcomplex

In order to examine the composition of the 185-kDa subcomplex observed in Figure 1 we again applied MS/MS. A single charge state isolated from this complex and subjected to collision with argon at an accelerating potential of 130 V yielded a well-resolved tandem mass spectrum, despite the low intensity of the subcomplex relative to the intact complex ( Figure 3A). Three series of ions are observed at the low m/z region ( Figure 3B). On the basis of their measured masses we assign these species to Rpn5, Rpn6, and Rpn9. Between 8,000 to 16,000 m/z, ions arising from the removal of a single subunit are observed ( Figure 3C). Although Rpn8 and the modified Rpn11 have similar masses, by simulating the charge states based on the known sequence of the subunits we can clearly distinguish between the two possibilities. We could then conclude that Rpn8 and not Rpn11 is a component of the subcomplex. These ions are therefore attributed to three different heterotrimers; Rpn5:6:8, Rpn5:8:9, and Rpn6:8:9.

**Figure 3:**
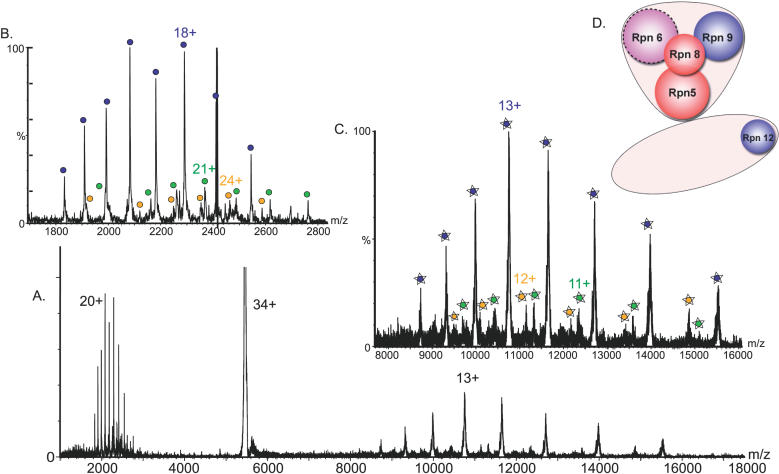
MS/MS of the Lid Subcomplex (A) Spectrum showing the dissociation of the 34+ charge state. (B) Expansion of the spectrum over the m/z range of 1,600–2,800. Three individual subunits are assigned Rpn5, Rpn6, and Rpn9 (orange, green, and blue circles, respectively). (C) Expansion of the 8,000–16,000 m/z region. Three different hetrotrimers are assigned: Rpn5:Rpn6:Rpn8, Rpn5:Rpn8:Rpn9, and Rpn6:Rpn8:Rpn9 (blue, green, and orange stars, respectively). By extrapolation we can conclude that the subcomplex is composed of four subunits, Rpn5, Rpn6, Rpn8, and Rpn9. Rpn8 is not observed in its monomeric form; however, because it exists in all three different hetrotrimers, we can conclude it adopts a central position within the subcomplex. (D) A schematic summarizes the structural data obtained from MS and MS/MS results. Red indicates core subunits, while purple and to a greater extent blue specifies peripheral subunits.

Interestingly, while Rpn8 is not detected as an individual subunit, it is present in all three heterotrimers. By extrapolation, therefore, we can conclude that prior to the collisional activation, the subcomplex is a tetramer composed of Rpn5, Rpn6, Rpn8, and Rpn9. From the dissociation pattern generated by the MS/MS experiment we can determine that Rpn8 occupies a central position within this subcomplex ( Figure 3D). It is noteworthy that the two charge series assigned to monomeric Rpn9 and its stripped heterotrimer, namely Rpn5:Rpn6:Rpn8, are the most intense series in the spectra. This is further evidence for the ease of dissociation of Rpn9, implying a relatively weak interaction with neighboring subunits.

### Chemical Cross-Linking Analysis

The cross-linking agent BS ^3^ is a bifunctional reagent linking amines, and is selective for the N-terminal amino group and lysine side chains, with an eight-carbon spacer arm of 11.4 Å. Denaturing gel electrophoresis before and after chemical cross-linking [ 44] shows that the reaction resulted in the production of covalently linked species labeled A to F according to their electrophoretic mobility ( Figure 4A). The bands were assigned as described in Materials and Methods ( Table 2). As an example, Figure 4B shows part of the matrix-assisted laser desorption/ionization (MALDI) mass spectrum of the peptide mixture from band D containing Rpn3, Rpn5, and Rpn8. The inset within the figure illustrates the MS/MS analysis of one of the peptides corresponding to Rpn5.

**Figure 4:**
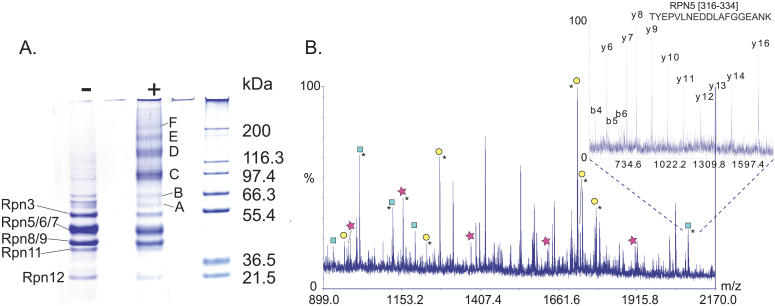
Chemical Cross-Linking of the Lid Complex (A) Bis-Tris 4%–12% denaturing gel, stained with Colloidal Blue, enables separation of proteins before (−) and after (+) cross-linking revealed six new bands, A-F. The absence of Sem1 in this analysis is attributed to its low molecular mass and is consistent with other analyses [ 10, 25, 56]. (B) A MALDI-TOF/TOF spectrum of trypsin digestion of band D. Three proteins were identified within this band: Rpn3 (yellow), Rpn5 (cyan), and Rpn8 (pink). Peaks labeled by asterisks were sequenced in order to identify the peptide unambiguously. The inset shows a low-energy collision-induced dissociation of an Rpn5 peptide producing mainly C-terminal (y) sequence ions.

**Table 2:**
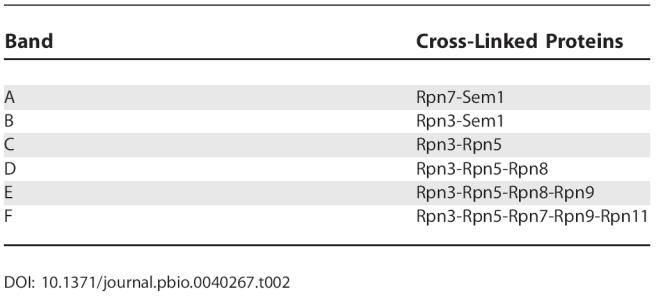
Cross-Linked Subunits Obtained from Bands A–F

Several conclusions can be drawn from the information extracted from the six cross-linked bands. As Sem1 is present in two bands cross-linked with Rpn7 and with Rpn3, we can assume that Sem1 interacts with both subunits. Moreover, due to the small molecular mass of Sem1, it is reasonable to assume that Rpn3 and Rpn7 are in close proximity to one another. In band C, Rpn3 is observed accompanied by Rpn5, and we can therefore conclude that Rpn3 binds to Rpn5. In addition, the subunits identified in bands D and E further confirm the formation of the subcomplex comprising Rpn5, Rpn8, and Rpn9 identified from our intact complex data. For Rpn11 (band F) we can deduce that it binds either directly to Rpn5:9 or is anchored by Rpn3:7 to Rpn5:9. In summary, cross-linking analysis allows determination of four additional interactions within the lid.

## Discussion

We acquired remarkably well-resolved mass spectra of the intact lid from S. cerevisiae despite the fact that we anticipated that the nine-component endogenous complex would be heterogeneous. From our data we were able to determine the subunit composition of the intact complex, the substoichiometric complexes present in solution, and those generated as a result of our MS/MS approach. Together with chemical cross-linking, our MS approach allowed us to obtain precise additional information regarding the subunit organization of the lid complex. Based on our data we constructed an interaction map of the lid subunits ( Figure 5). We identified a heterotetrameric core structure formed by Rpn5, Rpn6, Rpn8, and Rpn9, in which Rpn8 is centered. The absence of Rpn11 from this complex shows that it forms after purification and indicates that it is a stable subcomplex in solution. In addition, we revealed that Sem1 binds to both Rpn7 and Rpn3. Considering the small size of Sem1, we assume that Rpn3 and Rpn7 are also interacting. We could also determine that Rpn3 binds Rpn5. Interestingly, we determined a substoichiometric complex of the lid in which Rpn6 is absent. Close inspection of the spectrum suggests that 20%–30% of the purified lid complexes lack Rpn6. The MS/MS results clearly show that both Rpn9 and Rpn12 and, to a lesser extent, Rpn6, readily dissociate from the lid complex. It is established that the extent of interaction between protein subunits as well as their exposure underlies the release of subunits in tandem MS of heteroligomeric complexes [ 45]. This indicates that these subunits are therefore located at the periphery of the complex. Consistent with this, Rpn9 is known to be a nonessential subunit of the lid [ 10]. Interestingly, it has been shown previously that Rpn10, which stabilizes the association of the lid and base, interacts with both Rpn9 and Rpn12 [ 20]. This suggests that Rpn9 and Rpn12 are located near the contact surface between the base and the lid.

**Figure 5:**
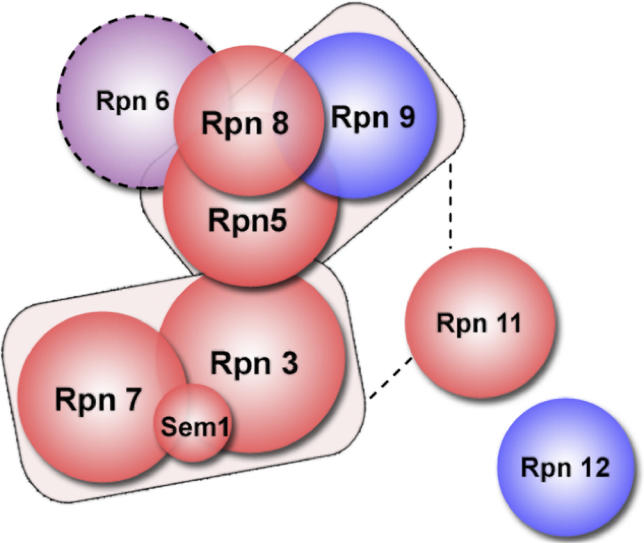
An Interaction Map Summarizing the Data Obtained from Both Native MS and Cross-Linking MS Analysis Rpn5, Rpn6, Rpn8, and Rpn9 form a tetrameric structure, in which Rpn8 occupies a central position. The dashed line circling Rpn6 indicates that it is present in substoichiometric amounts. Both Rpn7 and Rpn3 bind to Sem1. Considering the small size of Sem1, it is reasonable to suggest that Rpn3 and Rpn7 are in close proximity. In addition, we could determine that Rpn3 binds to Rpn5. Rpn11 interacts with Rpn3:7 or Rpn5:9; these possible interactions are labeled with a dashed line. No Interactions were determined for Rpn12. Subunits are colored either red, purple, or blue, indicating their increasing exposure within the complex.

A very recent study has shown that an *rpn6* temperature-sensitive mutant grown at restrictive temperatures contained a subcomplex comprising four out of the nine lid components, Rpn5, Rpn8, Rpn9, and Rpn11 [ 26]. A similar study with an *rpn7* temperature-sensitive mutant demonstrated the presence of an Rpn5:6:8:9:11 subcomplex [ 25]. From our cross-linking data, we could not conclude whether Rpn3:7 or Rpn5:9 were anchored to Rpn11. However the results from the *rpn6* and *rpn7* mutants support the scenario in which Rpn5:9 recruits Rpn11 into the Rpn5:6:8:9:11 subcomplex. In addition, the *rpn7* mutation prevented the incorporation of Rpn3, Rpn7, and Rpn12 into the lid. These results, together with the evidence that Rpn5:6:8:9 exists as a stable independent subcomplex in solution ( Figure 1), are consistent with the Rpn5:6:8:9 subcomplex forming the scaffolding core of the lid.

Taking together our data with existing data from analysis of mutants [ 25, 26] and results of previous two-hybrid analysis [ 20, 22, 23, 46] ( Table 3), we propose a detailed interaction map ( Figure 6). The fact that all the data could be integrated into a single model increases our confidence in the MS analysis. In the model, two clusters become apparent. The first structural cluster includes Rpn5, Rpn6, Rpn8, Rpn9, and Rpn11, in agreement with the cluster suggested previously [ 20]. Within the second cluster Rpn3, Rpn7, Rpn12, and Sem1 are included, in accord with recent observations [ 25, 26]. The link between the two subcomplexes is between Rpn5:Rpn3, and not Rpn7 as suggested by the analysis of an *rpn7* mutant [ 25]. These results explain previous observations in Schizosaccharomyces pombe that in cells in which *rpn5* is deleted the 26S proteasomes misassemble [ 47]. Moreover, our structural organization indicates that Rpn11 forms extensive interactions with all three subunits, Rpn5, Rpn8, and Rpn9. Furthermore, in our model Rpn6 is opposite Rpn9 and Rpn12. As these may interact with Rpn10 at the lid–base junction, it follows that Rpn6 is exposed to the cellular environment.

**Table 3:**
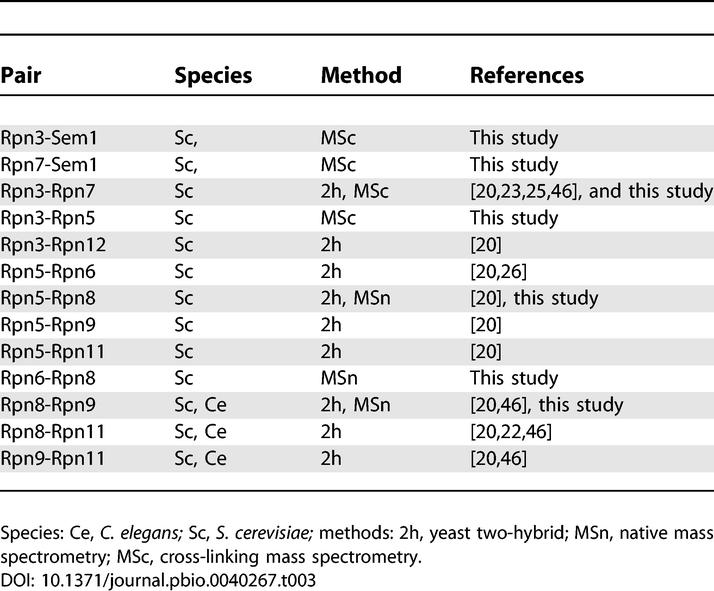
Subunit–Subunit Interactions within the 19S Lid from Saccharomyces cerevisiae

**Figure 6:**
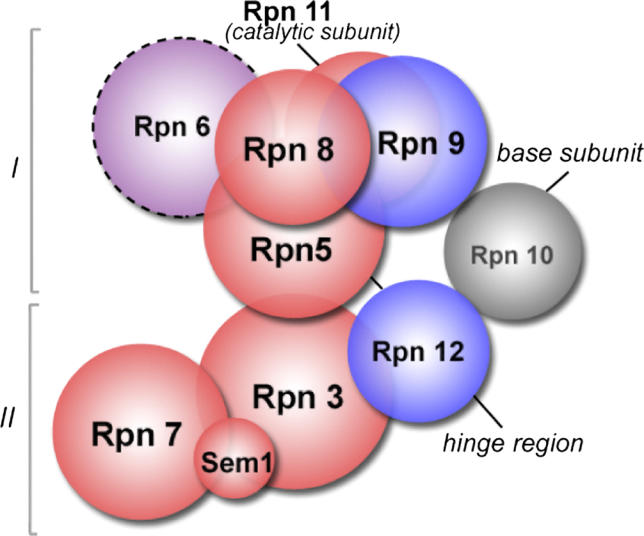
Structural Organization of the 19S Lid A plot summarizing the interactions within the lid, based on interactions identified here in combination with those determined previously. The dashed line circling Rpn6 indicates that it is present in substoiciometric amounts. Rpn10 (in gray) is added to the model to emphasize the orientation of the complex relative to the base and the cellular environment. The two apparent clusters are labeled. Subunits are colored either red, purple, or blue, indicating their increasing exposure.

Given the significant genetic similarity shared between the lid and the COP9 signalosome, we investigated their structural homology. Table 4 summarizes the protein–protein interactions identified within the CSN subunits. Out of the 21 interactions detected, nine were observed among the 19S lid complex ( Figure 6), and eight of the interactions, although not physically recognized, are feasible with our current model, while 4 interactions are not in correspondence with the lid structure. We speculate that the dense web of protein–protein interactions detected within the CSN complex could also be due to the tendency of the two-hybrid system to produce false positives [ 22]. However, it is interesting to note that six of the nine similar interactions and two of the eight feasible interactions are found within the Rpn5, Rpn6, Rpn8, Rpn9, and Rpn11 cluster. It is also worth noting that a 2-D electron microscopy study [ 48] did not deduce a common architecture for the two complexes. However, since both particles have similar sizes, show asymmetric arrangements of their subunits, possess a central channel, and share a large number of similar interactions, the two particles are likely to possess some common structural features.

**Table 4:**
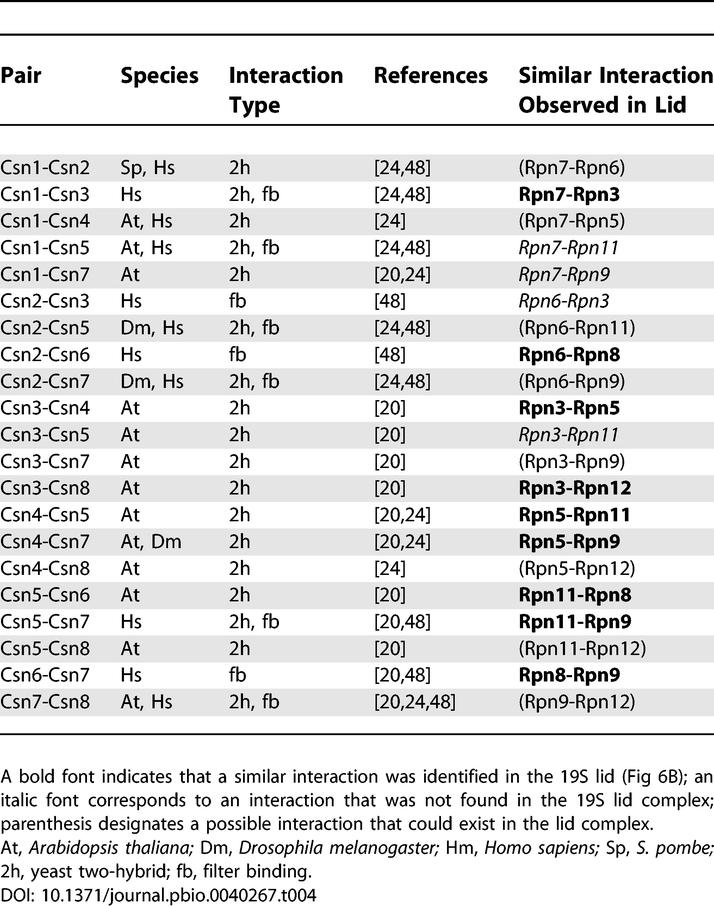
Subunit–Subunit Interactions within the COP9

The major function of the lid is determined by Rpn11, a specialized isopeptidase that tightly couples the deubiquitination and degradation of substrates [ 5– 7]. So far, no catalytic activity has been described for any other subunit. A recent study suggests that the role of these subunits is of a scaffold for the other binding partners [ 49], namely Rpn11, and the base subunits as well as the proteolyic substrates and soluble cofactors. Interestingly, Rpn11 is unable to fold as an isolated subunit into a native, active conformation [ 6]. Neighboring subunit interactions are probably required to position conjugates for cleavage by Rpn11. The multiple protein–protein interactions observed in this study for S. cerevisiae Rpn11 are in accordance with this assumption.

Given that the only interaction observed linking the two protein clusters in the lid is between Rpn3 and Rpn5, this implies that there is a flexible hinge region which allows movement necessary for productive interaction of substrates with Rpn11. Taking into consideration the fact that Rpn6 is present at substoichiometric amounts, and that Rpn11 forms extensive protein interactions, it is interesting to speculate that after substrate binding, Rpn6 dissociates to expose active Rpn11. Support for our hypothesis comes from the finding that Rpn6 is essential for assembly of the lid; however, *rpn6* mutants do not affect the activity of 26S proteasome after assembly [ 26]. This may imply that Rpn6 acts as a fail-safe mechanism, at least in *S. cerevisiae,* ensuring that active sites are exposed only after assembly is completed. Given the high natural abundance of proteasome particles in the cell and the need to prevent indiscriminate proteolysis of proteins, the masking of active sites before full assembly could be an essential regulatory mechanism. We anticipate that further examination of the levels of Rpn6 within the 19S lid complex will validate this scenario.

In summary, the results presented here extend significantly previous models based on pairwise interactions and allow interesting comparison with the structure of the signalosome. Our model is consistent with a core assembly of four subunits that provides a scaffold for recruitment of additional subunits. Also apparent from our analysis is the fact that subunits are assembled into two protein clusters, possibly providing a cleft in which a polyubiquitinated substrate can be accommodated. Based on our finding of substoichiometric quantities of Rnp6, and its position within the complex, we speculate that a regulatory mechanism involving dissociation of Rpn6 is responsible for the exposure of the active site of Rpn11. Overall, therefore, the subunit architecture that we have described will not only benefit further structural analysis but has also provided insight into the function and regulation of the 19S lid.

More generally, the results of this study highlight major advantages over existing approaches for generating interaction maps. For example, it is established that the yeast two-hybrid approach is complicated by the fact that protein interactions are queried pairwise. Interactions of subunits within protein complexes, such as the anaphase-promoting complex, can be underrepresented in two-hybrid datasets because assembly of such complexes may require higher-order interactions between multiple subunits. A second potential problem is that endogenous proteins may facilitate two-hybrid interactions that are not direct, and the presence of endogenous copies of proteins may confuse the analysis [ 22]. The other existing approach that has been widely applied is to generate mutants for functional analysis of this complex [ 25, 26]. This method can also be limited by the availability of appropriate mutants, lack of knowledge about how to generate appropriate mutants, and interpreting the effects of the mutations. The fundamental advantage of our approach is that it is carried out with the wild-type endogenous complex, and heterogeneity and subcomplex formation are immediately apparent from the spectra. Moreover, when combined with gas-phase dissociation we are able to probe the composition of protein subcomplexes and reveal peripheral subunits leading to definition of the structural organization of the complex. The fact that the assembly state of this asymmetric complex, with multiple distinct subunits, can be determined and results incorporated into a comprehensive model highlights the tremendous potential of MS. We anticipate that this approach will be used for determining the organization of many other important cellular complexes for which very little structural data exists.

## Materials and Methods

### Materials.

Bis(sulfosuccinimidyl) (BS ^3^) suberimidate was purchased from Pierce Biotechnology (Rockford, Illinois, United States). Modified trypsin and RNasin were obtained from Promega (Madison, Wisconsin, United States). Centricon YM10 centrifugal filters were purchased from Millipore (Billerica, Massachusetts, United States) and Nanosep centrifugal devices were purchased from Pall (East Hills, New York, United States). Other reagents and solvents used were analytical reagents or high-performance liquid chromatography grade where appropriate, and were purchased from Sigma-Aldrich (Saint Louis, Missouri, United States).

### Protein expression and purification.

Plasmid pJS-TM53H [ 50] was modified by overlap PCR to replace the Myc epitopes with 12 histidine codons, resulting in plasmid pT2H12. The S. cerevisiae RJD415 strain *(MATa can1–100 leu2–3,112 his3 trp1–1 ura3–1 ade2–1 pep4::TRP1 bar1::LEU2)* was transformed with a PCR product from a reaction with pT2H12 as the template using a forward primer homologous to the 42 nucleotides preceding the stop codon of *RPN11,* and a reverse primer with 41 nucleotides that are homologous to the region following the stop codon. The last 21 nucleotides of the forward and reverse primers are respectively homologous to the first and last regions of the tagging cassette of pT2H12. Transformants were selected on synthetic dropout–HIS agar plates. Integrants were screened for by nickel precipitation using Ni-NTA Agarose (Qiagen, Valencia, California, United States) followed by Western blot analysis of the eluates with anti-His and anti-Pad1 ( S. pombe Rpn11) antibodies. A positive transformant was selected and designated as RJD2909.

The RJD2909 strain was grown in yeast/peptone/dextrose (YPD) medium, and levels of Rpn11 ^H12^ were monitored through late stationary phase by nickel precipitation and detection by anti-His antibodies. Expression levels of Rpn11 ^H12^ remained constant through all phases of growth. A large (170 l) growth of RJD2909 in YPD was performed at the UCLA Fermentor Facility. The cells were harvested at an OD _600_ of 18.4 yielding a 1.7 kg cell pellet and were frozen at −80 °C.

A cell pellet of 70 g was thawed in 140 ml of chilled lysis buffer (25 mM Tris [pH 7.5], 200 mM NaCl, 20 mM imidazole, 10 mM β-mercaptoethanol, 0.3% Triton X-100, 50 mM NaF, 1 mM PMSF) and 3 complete EDTA-free protease inhibitor cocktail tablets (Roche, Indianapolis, Indiana, United States), and added to 200 ml of chilled 500-μm glass beads inside a stainless steel chamber of a BeadBeater (BioSpec Products, Bartlesville, Oklahoma, United States). The chamber jacket was filled with ice water, and the BeadBeater was run for 1 min and allowed to cool for 1 min for six cycles at 4 °C. The contents of the chamber were applied with vacuum to a filter unit (Nalgene, Rochester, New York, United States) equipped with a 180-μm nylon net filter (Millipore) to separate the glass beads from the supernatant. The filtrate was subsequently spun at 5,000 rpm in an Eppendorf 5804 centrifuge (Eppendorf, Westbury, New York, United States) for 20 min to remove intact cells and precipitated material.

The cell lysis solution was applied to a column of Ni-NTA Superflow resin (Qiagen) at a flow rate of 2 ml/min. The resin was subsequently washed in wash buffer (25 mM Tris [pH 7.5], 500 mM NaCl, 0.3% Triton X-100, 50 mM imidazole, 10 mM β-mercaptoethanol) and starting buffer (25 mM Tris [pH 7.5], 200 mM NaCl, 20 mM imidazole, 10 mM β-mercaptoethanol), and the bound protein was eluted with elution buffer (25 mM Tris [pH 7.5], 100 mM NaCl, 300 mM imidazole, 10 mM β-mercaptoethanol). Peak fractions were collected and concentrated to a final volume of ≥2 ml by ultracentrifugation in a Centricon concentrator (Millipore), with a molecular weight cutoff of 100 kDa.

The sample was loaded and run on a 26:60 Sephacryl S-400 gel filtration column (Amersham Biosciences, Little Chalfont, United Kingdom) at a flow rate of 0.5 ml/min with gel filtration buffer (50 mM Tris [pH 7.5], 200 mM NaCl, 5% glycerol, 1 mM DTT). The lid particle elutes in three peaks at 156 ml (peak 1), 164 ml (peak 2), and 196 ml (peak 3). Based on calculations derived from calibration standards, the theoretical molecular masses of the three peaks are 1,800 kDa for peak 1, 200 kDa for peak 2, and 500 kDa for peak 3. In peaks 1 and 2, the lid particle elutes as part of the 19S proteasome regulatory particle. In peak 3, the lid particle elutes in a relatively pure form. Fractions corresponding to peaks 2 and 3 were concentrated to ∼200 μl by ultracentrifugation in an Amicon Ultra-15 concentrator (Millipore) with a molecular weight cutoff of 100 kDa. The protein concentrations of peak 2 and 3 were 4 mg/ml and 10 mg/ml, respectively, as determined by a DC protein assay (Bio-Rad, Hercules, California, United States).

### MS conditions.

To confirm the protein composition of the lid complex the sample was analysed by established proteomics methods using a high-resolution MALDI time of flight (TOF)/TOF (Applied Biosystems, Foster City, California, United States), and a C18 75 μm × 15 cm column on an UltiMate capillary system (Dionex, Sunnyvale, California, United States) connected to a Probot fraction collector (Dionex). All 9 subunits were identified by automated MALDI-TOF/TOF peptide mapping and sequence database searching of S. cerevisiae proteins. Electrospray ionization MS and MS/MS experiments were conducted on a high mass quadrupole TOF–type instrument [ 42, 51] adapted for a QSTAR XL platform, [ 42, 51]. In this instrument a flow-restricting sleeve is installed in the front part of the Q _0_ to increase the pressure locally and allow collisional cooling of heavy ions. In addition, the low-frequency extended mass range of Q _1_ permits the isolation of ions up to 35,000 m/z.

For analysis of the intact complex, prior to mass spectrometry 25 μl of the 25 μM sample of the lid complex was buffer exchanged using a Nanosep centrifugal device with a molecular weight cutoff of 10 kDa into 1 M ammonium acetate. For accurate mass determination of the individual protein subunits, the sample was washed and eluted from a C _4_ ZipTip column (Millipore) under denaturing conditions (50% acetonitrle and 0.1% formic acid). All other mass spectra were recorded from aqueous solution conditions in 1 M ammonium acetate. Typically, 1.5 μl of solution was electrosprayed from gold-coated borosilicate capillaries prepared in-house as described [ 52]. The following experimental parameters were used: capillary voltage up to 1.2 kV; declustering potential, 150 V; focusing potential, 250V; second declustering potential, 55V; and collision energy up to 200 V, microchannel plate 2350 V. In MS/MS the relevant m/z value was selected in the quadrupole and argon was used as a collision gas at maximum pressure. All spectra were calibrated externally by using a solution of cesium iodide (100 mg/ml). Spectra are shown here with minimal smoothing and without background subtraction.

### Assignment of peaks in mass spectra.

The masses of the individual subunits and the complexes were calculated from the spectra according to a method in which the charge is iterated over the measured mass value to determine the best fit to the experimental data [ 53]. The accurate masses of the individual subunits were determined from a denatured solution of the complex. The molecular masses of Rpn5, Rpn6, Rpn7, Rpn8, Rpn9, Rpn11, and Rpn12 ( Table 1) are consistent with those reported in the database and consistent with the N-terminal modifications previously reported [ 54]. We could also clearly identify that the initiator methionine is removed and the N-terminal amino group is acetylated in the Sem1 subunit. In addition, from the mass measured for Rpn3 we could determine that the first 16 residues of this subunit are truncated.

### Cross-linking reactions and tryptic digestion.

Solutions of the cross-linking reagent BS ^3^ were prepared immediately before use at a concentration of 10 mM in buffer (200 mM NaCl, HEPES 50 mM [pH 7.5]). The cross-linking reaction was performed with a lid concentration of 4.8 μM and BS ^3^ ratio of 1:50 for 2 h on ice. The reaction was terminated by addition of 2 M Tris-HCl (pH 7.4) to give a final concentration of 50 mM Tris-HCl. SDS-PAGE gel analysis was performed on Bis-Tris 4%–12% gels using the Novex Colloidal Blue kit (Invitrogen, Carlsbad, California, United States). For analysis of gel bands by in-gel digest, proteins were digested with 2% trypsin wt/wt at 37 °C for 12 h in 50 mM ammonium bicarbonate [ 55].

### MS/MS analysis of cross-linked peptides.

The digestion mixture was subjected to a Nano LC column coupled to a Probot MALDI spotter followed by MS and MS/MS analysis using air as collision gas on a MALDI-TOF/TOF instrument (Applied Biosystems). Resolution on this instrument was within 30 ppm and MS data were acquired using software from Applied Biosystems. Typically, 5,000 spectra were acquired for each sample in MS or each MS/MS experiment. Throughout the assignment a set of four conditions were applied: (1) the peak intensity had to be ≥ 10%; (2) only peptides identical to those observed in the absences of cross-linking reagent were considered; (3) each protein had to be represented by at least four independent peptides; and (4) peptides for which there was more than one amino acid composition possible had to be sequenced. The assignment was validated further by analysis of the MALDI-MS/MS peptide sequencing spectra. Each protein had at least one peptide confirmed by the MALDI-TOF/TOF sequence analysis. It was not practical to apply these conditions to Sem1 due to its low molecular weight (10 kDa). The presence of Sem1 was confirmed by identifying two Sem1 peptides and sequencing by MALDI-MS/MS spectra.

Using our set of criteria for assignment, proteins identified in bands A–F were considered cross-linked. In the case of band A three proteins, Rpn3, Rpn7, and Sem1, were identified. Considering the apparent migration mass for this band, the only combination of proteins consistent with this mass was Rpn7-Sem1. Therefore, the presence of Rpn3 is likely to arise from the two neighboring bands of native Rpn3 and band B. In all the other 5 bands the apparent masses deduced from migration on the gel were within error, taking into account changes in migration anticipated from cross-linking [ 44], and were therefore entirely consistent with the cross-linked protein components.

## Abbreviations

CSN: COP9 signalosome
MALDI: matrix-assisted laser desorption/ionization
MS: mass spectrometry
MS/MS: tandem mass spectrometry
TOF: time of flight
